# The *Plasmodium* alveolin IMC1a is stabilised by its terminal cysteine motifs and facilitates sporozoite morphogenesis and infectivity in a dose-dependent manner

**DOI:** 10.1016/j.molbiopara.2016.09.004

**Published:** 2017-01

**Authors:** Fatimah S. Al-Khattaf, Annie Z. Tremp, Amira El-Houderi, Johannes T. Dessens

**Affiliations:** Pathogen Molecular Biology Department, London School of Hygiene & Tropical Medicine, Keppel street, London WC1E 7HT, United Kingdom

**Keywords:** Alveolin, Intermediate filament, Cytoskeleton, Palmitoylation, Sporozoite, *Plasmodium berghei*

## Abstract

Apicomplexan parasites possess a unique cortical cytoskeleton structure composed of intermediate filaments. Its building blocks are provided by a conserved family of proteins named alveolins. The core alveolin structure is made up of tandem repeat sequences, thought to be responsible for the filamentous properties of these proteins. A subset of alveolins also possess conserved motifs composed of three closely spaced cysteine residues situated near the ends of the polypeptides. The roles of these cysteine motifs and their contribution to alveolin function remains poorly understood. The sporozoite-expressed IMC1a is unique within the *Plasmodium* alveolin family in having conserved cysteine motifs at both termini. Using transgenic *Plasmodium berghei* parasites, we show in this structure-function analysis that mutagenesis of the amino- or carboxy-terminal cysteine motif causes marked reductions in IMC1a protein levels in the parasite, which are accompanied by partial losses of sporozoite shape and infectivity. Our findings give new insight into alveolin function, identifying a dose-dependent effect of alveolin depletion on sporozoite size and infectivity, and vital roles of the terminal cysteine motifs in maintaining alveolin stability in the parasite.

## Highlights

• The terminal cysteine motifs of the alveolin IMC1a stabilise the protein.
• IMC1a depletion dose-dependently affects sporozoite morphogenesis.
• Sporozoite size correlates with sporozoite infectivity.

## Graphical abstract

**Figure:**
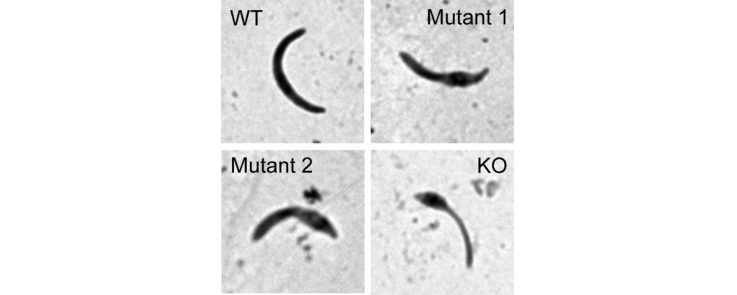

## 1 Introduction

*Plasmodium* species, the causative agents of malaria, have a complex life cycle in vertebrate host and mosquito vector. Among the many different developmental forms of the parasite feature three motile and invasive stages (also known as ‘zoites'): the ookinete, sporozoite and merozoite. The zoites of *Plasmodium*, as well as those of related apicomplexan parasites, possess an unusual cortical structure termed the pellicle. The pellicle is defined by a double membrane structure termed the inner membrane complex (IMC) situated directly underneath the plasma membrane, which is equivalent to a system of flattened sacs or alveoli [1], [2], [3]. On the cytoplasmic face of the IMC is anchored a network of intermediate filaments termed the subpellicular network (SPN), the function of which is to support the pellicle membranes and give the cell mechanical strength [4].

A family of proteins now termed alveolins have been identified as components of the SPN [4], [5]. The alveolin superfamily includes structurally related proteins from apicomplexan parasites, ciliates and dinoflagellate algae, the three phyla comprising the Alveolata superphylum [6]. In the genus *Plasmodium*, 13 conserved and syntenic alveolin family members have been identified that are differentially expressed among the three different zoites stages of malaria parasites [7], [8]. It has been shown in the rodent malaria species *P. berghei* that disruption of alveolins gives rise to morphological aberrations that are accompanied by reduced tensile strength of the zoite stages in which they are found [5], [8], [9], [10], [11]. In *Tetrahymena thermophila*, knockdown of the alveolin *Tt*ALV2 was also reported to affect cell morphology [12], indicating that alveolin functions, like their structures, are evolutionary conserved. *Plasmodium* alveolins also have roles in parasite gliding motility [5], [9], [10], [11] most likely by tethering glideosome associated proteins that reside in the IMC.

**Fig. 1:**
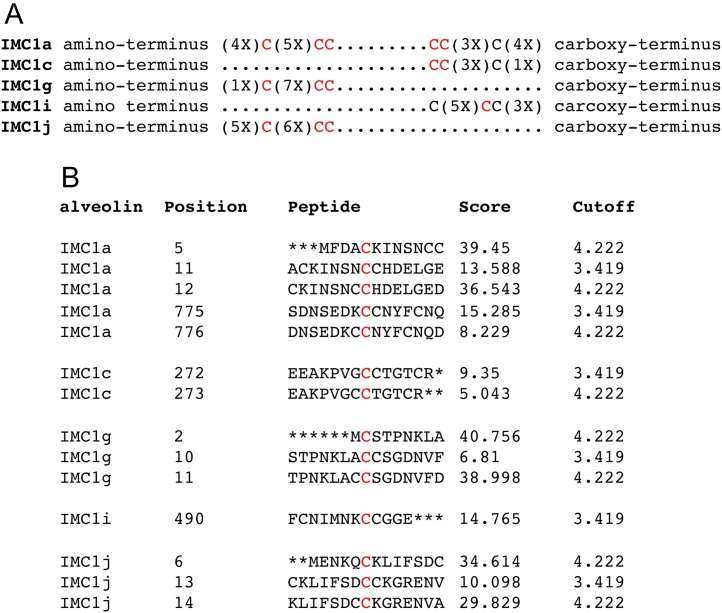
The *Plasmodium* alveolin cysteine motifs. A: Conserved cysteine motifs at the amino- and carboxy-terminal ends of *Plasmodium berghei* alveolins IMC1a (PbANKA_0402600), IMC1c (PbANKA_1202000), IMC1g (PbANKA_1240600), IMC1i (PbANKA_0707100) and IMC1j (PbANKA_1120400). The number of non-cysteine residues (X) adjacent to the conserved terminal cysteines (C) are indicated. Cysteines in red are predicted to be palmitoylated. B: Prediction scores of palmitoylated cysteine residues (red) using CSS-Palm 4.0 software (http://csspalm.biocuckoo.org/) and high threshold settings (95% specificity, 90% accuracy). (For interpretation of the references to colour in this figure legend, the reader is referred to the web version of this article.)

The alveolins identified in *Plasmodium* are characterised by having one or more highly conserved domains separated by regions of variable length and amino acid composition. These conserved ‘alveolin domains’ are composed of tandem repeat sequences [7], [12]. This has revealed an interesting parallel with metazoan intermediate filament proteins such as lamins and keratins, whose underlying architectures include a helical rod domain that can form coiled-coils by virtue of a seven amino acid tandem repeat structure [13]. These coiled-coil domains are thought to be fundamental for the filament-forming properties of these molecules. Apart from the conserved alveolin domains, a subset of the alveolins also possess conserved cysteine motifs close to their amino- or carboxy-terminus (Fig. 1). These motifs are made up of a single cysteine and a double cysteine that are separated by a small number of other amino acids (Fig. 1). With the exception of IMC1i, The N- and C-terminal motifs are inverted, with the single cysteine located nearest the end of the polypeptide (Fig. 1). The function of these cysteine motifs is largely unknown, although they have been suggested to provide sites for post-translational S-palmitoylation [14] (Fig. 1). A subset of alveolins in *Toxoplasma* (IMC1, IMC4, IMC14 and IMC15) possess similar conserved terminal cysteine motifs [14]. Because these conserved cysteine motifs have not been identified in alveolins from dinoflagellates or ciliates, their function could be related to the unique motility and/or cytokinesis associated with the Apicomplexa [6]. IMC1a is the only *Plasmodium* alveolin with conserved cysteine motifs at both ends, and in this study we employ site-directed mutagenesis and allelic replacement in *P. berghei* to investigate the contribution of these motifs to the function of the protein and the SPN as a whole. We also describe a new method for accurate size measurements of sporozoite populations, providing a valuable new tool for assessing sporozoite phenotypes.

## 2 Materials and methods

### 2.1 Animal use

All laboratory animal work is subject to regular ethical review by the London School of Hygiene and Tropical Medicine, and has approval from the United Kingdom Home Office. Work was carried out in accordance with the United Kingdom Animals (Scientific Procedures) Act 1986 implementing European Directive 2010/63 for the protection of animals used for experimental purposes. Experiments were conducted in 6–8 weeks old female CD1 mice, specific pathogen free and maintained in filter cages. Animal welfare was assessed daily and animals were humanely killed upon reaching experimental or humane endpoints. Mice were infected with parasites by intraperitoneal injection, or by infected mosquito bite on anaesthetized animals. Parasitemia was monitored regularly by collecting of a small drop of blood from a superficial tail vein. Drugs were administered by intraperitoneal injection or where possible were supplied in drinking water. Parasitized blood was harvested by cardiac bleed under general anaesthesia without recovery.

### 2.2 Parasite maintenance, transmission, culture and purification

*P. berghei* ANKA clone 2.34 parasites were maintained as cryopreserved stabilates or by mechanical blood passage and regular mosquito transmission. Mosquito infection and transmission assays were as previously described using *Anopheles stephensi* [5], [15] and infected insects were maintained at 20 °C at approximately 70% relative humidity.

### 2.3 Construction of gene targeting vectors

To allow mCherry tagging of IMC1a, an approximately 3.5 kb fragment corresponding to the entire *imc1a* gene (introns included) plus 5′-UTR was PCR amplified from *P. berghei* gDNA using primers pDNR-imc1a-F (ACGAAGTTATCAGTCGAGGTACCTTTCATGATTCTATCTATTGTTAATTTTAATTG) and pDNR-imc1a-R (ATGAGGGCCCCTAAGCTTTTATCTTGATTACAAAAATAATTACAACATTTG) and introduced into SalI/HindIII-digested pDNR-mCherry [16] by in-fusion to give plasmid pDNR-IMC1a/mCherry (Fig. S1).

To substitute the N-terminal cysteine motif of IMC1a (Mutant 1) primers IMC1a-Mut1-F (GAAAATAAATAGTAATCTCGAGCATGATGAGTTGGGAGAAGACA) and IMC1a-Mut1-R (ATTACTATTTATTTTCCATGCATCAAACATTTTAATTAAATG) were used to PCR amplify pDNR-IMC1a/mCherry, and the PCR product was circularized by in-fusion to give plasmid pDNR-IMC1a-Mutant 1. Introduction of a diagnostic XhoI site changes the double cysteine (CC) in the amino-terminal motif to leucine-glutamate (LE). To substitute the C-terminal cysteine motif of IMC1a (Mutant 2) primers IMC1a-Mut2-F (CTCGAGAATTATTTTTGGAATCAAGATAAAAGCTTAGGGGC) and IMC1a-Mut2-R (CCAAAAATAATTCTCGAGTTTGTCTTCAGAATTATCACTTTTTTTT) were used to PCR amplify pDNR-IMC1a/mCherry, and the PCR product was circularized by in-fusion to give plasmid pDNR-IMC1a-Mutant 2. Introduction of a diagnostic XhoI site changes the double cysteine (CC) in the carboxy-terminal motif to leucine-glutamate (LE). To substitute the double cysteine from the C-terminal cysteine motif of IMC1a (Mutant 3) primers IMC1a-Mut3-F (CTCGAGAATTATTTTTGTAATCAAGATAAAAGCTTAGGGGC) and IMC1a-Mut3-R (ACAAAAATAATTCTCGAGTTTGTCTTCAGAATTATCACTTTTTTTT) were used to PCR amplify pDNR-IMC1a/mCherry, and the PCR product was circularized by in-fusion to give plasmid pDNR-IMC1a-Mutant 3. This mutation introduces a diagnostic XhoI restriction site, changing the double cysteine (CC) in the carboxy-terminal motif to leucine-glutamate (LE). To substitute the single cysteine from the C-terminal cysteine motif of IMC1a (Mutant 4) primers IMC1a-Mut4-F (TTATTTCGCGAATCAAGATAAAAGCTTAGGGGC) and IMC1a-Mut4-R (TTGATTCGCGAAATAATTACAACATTTGTCTTCAGAATTATCACT) were used to PCR amplify pDNR-IMC1a/mCherry, and the PCR product was circularized by in-fusion to give plasmid pDNR-IMC1a-Mutant 4. This mutation introduces a diagnostic NruI restriction site, changing the single cysteine (C) in the carboxy-terminal motif to alanine (A) (Fig. S1).

Primers hDHFR/ERI-F (ACAAAGAATTCATGGTTGGTTCGCTAAACT) and hDHFR/ERI-R (ACCATGAATTCTTTGTAACATTTAGGTGTGTATTTATATATATAAGC) were used to PCR amplify plasmid pLP-hDHFR [17]. The PCR product was circularized by in-fusion, to give plasmid pLP-hDHFR/EcoRI. In this plasmid the BamHI restriction site at beginning of the hDHFR gene is replaced with an EcoRI recognition sequence. A 1.7 kb fragment corresponding to the *hdhfryfcu* gene was PCR amplified from plasmid pL0035 with primers hDHFRyFCU-F (ATGTTACAAAGAATTCATGGTTGGTTCGCTAAACTG) and hDHFRyFCU-R (AAGAAAAACGGGATCCTTAAACACAGTAGTATCTGTCACCAAAG) and introduced into EcoRI/BamHI-digested pLP-hDHFR/EcoRI by in-fusion to give pLP-hDHFRyFCU. A 0.75 kb fragment corresponding to the 3′UTR of the *imc1a* gene was amplified from *P. berghei* gDNA with primers pLP-imc1a-F (ATATGCTAGAGCGGCCAAAATATGGTATTTTAAAACTATTGAATTGG) and pLP-imc1a-R (CACCGCGGTGGCGGCCAGCGACACTTAAGAGATAGCATAAGA) and introduced into NotI-digested pLP-hDHFRyFCU by in-fusion to give plasmid pLP-hDHFRyFCU/IMC1a.

Cre-*lox*p recombination of pDNR-IMC1a/mCherry, pDNR-IMC1a-Mutant 1 and pDNR-IMC1a-Mutant 2, pDNR-IMC1a-Mutant 3 and pDNR-IMC1a-Mutant 4 was carried out with pLP-hDHFRyFCU/IMC1a to give the final targeting vectors pLP-IMC1a/mCherry-WT, pLP-IMC1a/mCherry-Mutant 1 to pLP-IMC1a/mCherry-Mutant 4, respectively.

Sequence verification across the IMC1a:mCherry-encoding region identified one targeting vector that had obtained an undesired frameshift close to the 5′-end of the *imc1a* coding sequence. This plasmid was used to generate a new IMC1a null mutant parasite (IMC1a/mCherry-KO) by the same targeting strategy as the other parasite lines.

### 2.4 Generation and genomic analysis of genetically modified parasites

Parasite transfection, pyrimethamine selection and dilution cloning were performed as previously described [18]. The *imc1a* gene targeting strategy employed double crossover homologous recombination, ensuring that its modifications were stable and non-reversible (Fig. S1). Prior to performing transfections, plasmid DNA was double-digested with KpnI and SacII to remove the plasmid backbone. Genomic DNA extraction was performed as previously described [15]. After transfection, drug resistant parasites were subjected to limiting dilution cloning. Integration of the selectable marker gene into the *imc1a* locus was confirmed by diagnostic PCR across the 3′ integration site using primers P3 (ACAAAGAATTCATGGTTGGTTCGCTAAAC) and P4 (TGCACACCCACCTGATTG) (Fig. S1). Integration of the mCherry-tagged IMC1a-encoding sequence into the *imc1a* locus was confirmed by diagnostic PCR across the 5′ integration site with primers P1 (GCACATTAATGCATTTGGG) and P2 (AACGGGATCTTCTAGTTACTTGTACAGCTCGTCCATGC) (Fig. S1). The absence of the unmodified *imc1a* allele in the clonal parasite lines was confirmed by diagnostic PCR with primers P1 and P4 (Fig. S1).

### 2.5 Sporozoite footprint measurements

Sporozoite-infected tissues were dissected from parasite-infected mosquitoes and the sporozoites gently released in a Dounce homogenizer. Sporozoites were spotted onto glass microscope slides, allowed to adhere and then air dried. After methanol fixation, Giemsa-stained images of individual cells were captured by microscopy on a Zeiss LSM510 inverted laser scanning confocal microscope. Using Zeiss LSM image browser software the circumference was measured, and the surface area occupied (i.e. the footprint) calculated. Statistical analysis was carried out using two-tailed *t*-test.

### 2.6 Western blot analysis

Parasite samples were heated directly in SDS-PAGE loading buffer at 70 °C for 10 min. Proteins were fractionated by electrophoresis through NuPage 4–12% Bis-Tris precast gels (Invitrogen) and transferred to PVDF membrane (Invitrogen) according to the manufacturer’s instructions. Membranes were blocked for non-specific binding in PBS supplemented with 0.1% Tween 20 and 5% skimmed milk for 1 h at room temperature. Rabbit polyclonal antibody against RFP (Abcam) diluted 1 in 5000 was applied to the membrane for 1 h at room temperature. After washing, membranes were incubated with goat anti rabbit IgG conjugated to horseradish peroxidase (HRP) (Abcam) diluted 1 in 5000 for 1 h at room temperature. After further washing, signal was detected by chemiluminescence (ECL western blotting substrate, Pierce) according to manufacturer’s instructions. For reprobing, the blot was incubated in 30% hydrogen peroxide solution for 30 min at 37 ° to inactivate residual HRP [19]. The membrane was reblocked and then incubated with monoclonal antibody 3D11 against circumzoite protein (CSP) diluted 1 in 1000 for 1 h at room temperature. After washing secondary goat-anti-mouse polyclonal antibody conjugated to HRP (Invitrogen 81–6520) diluted 1 in 5000 was added and incubated for 1 h at room temperature prior to washing and chemiluminescence detection.

### 2.7 RT-PCR analysis

Twenty midguts were harvested from parasite-infected mosquitoes at two weeks post-infection, pooled, and total RNA was extracted using a Qiagen RNeasy mini kit according to manufacturer's instructions. First strand cDNA was synthesized with M-MLV reverse transcriptase, (RNase H minus point mutation; Promega) using oligo(dT)25 as primer, for 1 h at 50 °C. Excess primer was removed by column purification (Qiaquick gel extraction kit; Qiagen) and the eluted cDNA was subjected to PCR amplification with primers A30 (ATATAGTCCATTTAGTTAGAGTTTGTG) and pDNR-imc1a-R (ATGAGGGCCCCTAAGCTTTTATCTTGATTACAAAAATAATTACAACATTTG) to amplify imc1a, and primers tub1-F (GAAGTAATAAGTATACATGTAGG) and tub1-R (ACACATCAATGACTTCTTTACC) to amplify tubulin 1.

### 2.8 Microscopy

For assessment of fluorescence, live parasite samples were assessed, and images captured, on a Zeiss LSM510 inverted confocal microscope and Zeiss LSM image browser software. For comparison of samples, images were captured with the same settings using the ‘reuse' function.

## 3 Results

### 3.1 The terminal cysteine motifs of IMC1a affect protein stability

To study expression and localization of IMC1a and variants of it in the parasite, we first generated a transgenic *P. berghei* line that expresses full-length IMC1a fused to a carboxy-terminal mCherry tag (Fig. S1), named IMC1a/mCherry-WT. To study the contribution of the cysteine motifs to IMC1a function, mutations substituting the three cysteines were introduced by site-directed mutagenesis removing either the N-terminal motif (named IMC1a/mCherry-Mutant 1) or the C-terminal motif (named IMC1a/mCherry-Mutant 2) (Fig. S1). The mutations introduced a diagnostic XhoI restriction site in order to screen targeting vectors and transgenic parasites for the presence of the desired mutation. Introduction of this XhoI site changes the double cysteine (CC) to a leucine-glutamate (LE) (Fig. S1). In addition, an IMC1a/mCherry targeting vector that contained a frame shift very near the 5′ end of the coding sequence was used to generate a new IMC1a null mutant parasite line (named IMC1a/mCherry-KO) using the same genetic approach as the other IMC1a lines (Fig. S1).

**Fig. 2:**
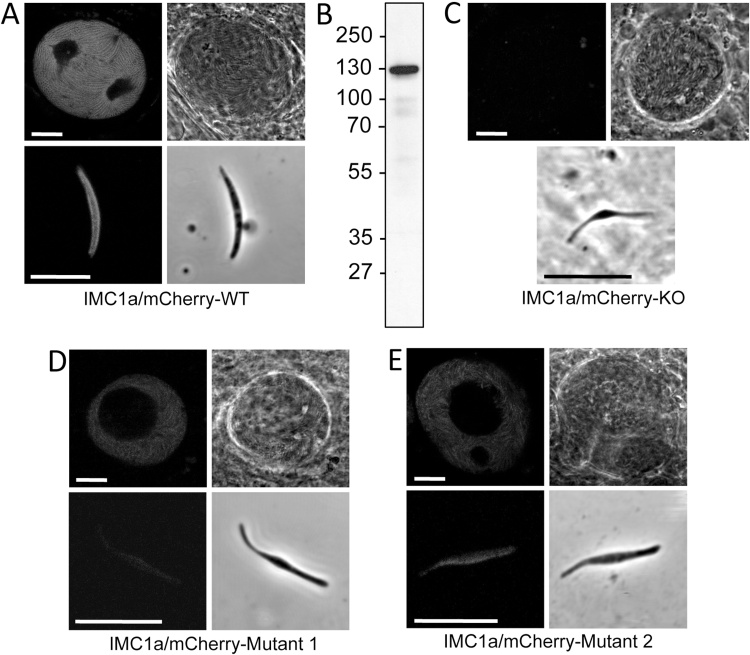
Phenotypic analyses of mCherry-tagged *PbI*MC1a parasite lines. A: Confocal fluorescence and brightfield images of a sporulating oocyst and sporozoite of parasite line IMC1a/mCherry-WT. B: Western blot of a sporozoite lysate of parasite line IMC1a/mCherry-WT (approximately 100,000 sporozoites loaded) using anti-mCherry antibodies, visualising the IMC1a:mCherry fusion protein. Size markers are shown on the left hand side. C–E: Confocal images of a sporulating oocyst and sporozoite of mutant parasite lines IMC1a/mCherry-KO (C); IMC1a/mCherry-Mutant 1 (D**)**; IMC1a/mCherry-Mutant 2 (E). Confocal images were captured using the same confocal microscope settings. Scale bars represent 10 μm.

Parasite line IMC1a/mCherry-WT developed normally in mouse and mosquito, and was readily transmitted by sporozoite-infected mosquito bite, demonstrating that the mCherry tag did not interfere with normal IMC1a function. Using fluorescence microscopy, strong mCherry-based fluorescence was detected in sporulating oocysts (Fig. 2A). In both midgut and salivary gland sporozoites, the fluorescence was concentrated at the cell periphery (Fig. 2A) consistent with the location of the SPN. Western blot analysis of sporozoite lysates using anti-mCherry antibodies detected one major band migrating at approximately 130 kDa, corresponding to the IMC1a:mCherry fusion protein (Fig. 2B). Some smaller proteins of much lower intensity were also detected probably resulting from low level proteolytic processing/degradation. As expected, IMC1a/mCherry-KO parasites did not exhibit mCherry fluorescence in oocysts or sporozoites (Fig. 2C), because the mCherry tag is not expressed due to an upstream frameshift. IMC1a/mCherry-KO oocysts displayed a phenotype comparable to that previously described for IMC1a null mutants [5], producing numbers of sporozoites similar to its WT counterpart (mean midgut oocysts/sporozoites per mosquito IMC1a/mCherry-WT: 43/15,000; IMC1a/mCherry-KO: 52/17,000; n = 10), but with abnormal size and shape (Fig. 2C).

**Fig. 3:**
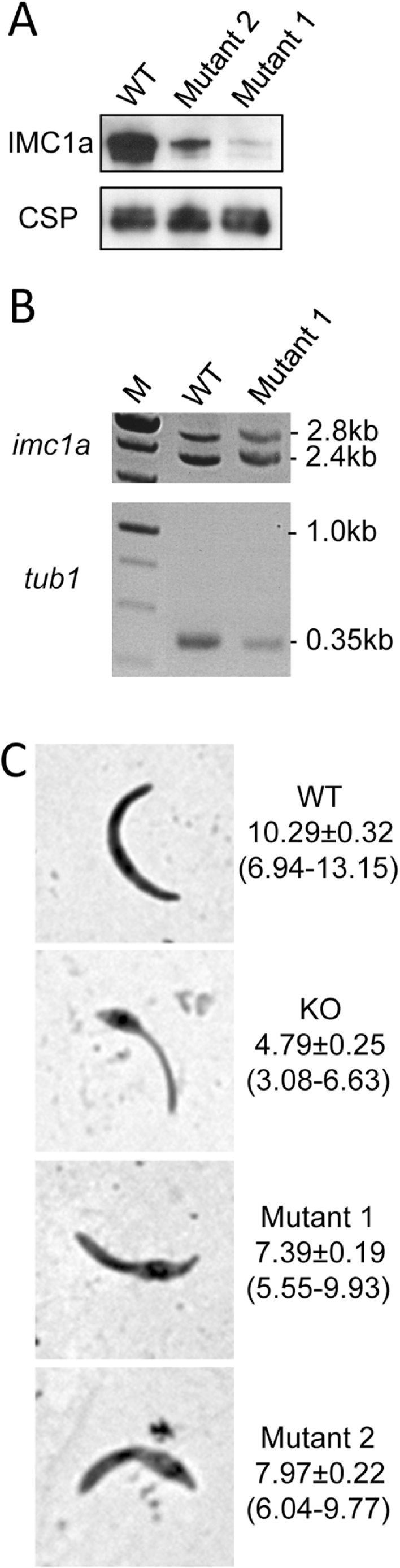
Cysteine mutations of IMC1a affect protein stability and sporozoite size. A: Western blot of sporozoite lysates from parasite lines IMC1a/mCherry-WT, Mutant 1 and Mutant 2, using anti-mCherry antibodies (*top*), or anti-CSP antibodies (*bottom*). B: Reverse transcription PCR analysis of *imc1a* mRNA levels (relative to *tub1* mRNA) in sporulating oocysts of parasite lines IMC1a/mCherry-WT and Mutant 1. C: Representative Giemsa-stained sporozoite images from parasite lines IMC1a/mCherry-WT, KO, and Mutants 1–2. Numbers give mean ± sem footprint measurements in μm^2^ and numbers in brackets give footprint range in μm^2^ (n = 27).

Parasite lines IMC1a/mCherry-Mutant 1 and Mutant 2 also displayed mCherry-based fluorescence in mature oocysts and sporozoites (Figs. 2DE), as expected, demonstrating that the full-length IMC1a protein was expressed. However, in both mutants the fluorescence levels were markedly lower compared to parasite line IMC1a/mCherry-WT (Fig. 2A). These observations indicated that the removal of the amino- or carboxy-terminal cysteine motif from IMC1a had adversely affected either the amount of the alveolin in the parasite, or the ability of its mCherry moiety to fluoresce. To distinguish between these possibilities, we quantified the amounts of IMC1a in sporozoite samples of the different parasite lines by western blot analysis. The values obtained revealed that, relative to circumsporozoite protein (CSP), significantly reduced amounts of IMC1a:mCherry fusion protein were present in Mutant 1 and Mutant 2 (on average 10% and 21% of WT levels, respectively; P < 0.001; n = 3) (Fig. 3A). These reduced levels of IMC1a:mCherry fusion protein in the parasite explain the lower fluorescence levels observed in Mutant 1 and Mutant 2. Sporozoites of IMC1a/mCherry-Mutant 1 had consistently lower levels of IMC1a than Mutant 2, although the differences were not statistically significant (P = 0.09).

Because all the transgenic parasite lines used in this study were generated with the same gene targeting strategy, and express IMC1a from the same, native *imc1a* promoter (Fig. S1), *imc1a* gene expression at the transcription level should not be affected. To confirm this, we carried out reverse transcription-PCR analysis on sporulating oocysts. This was done with Mutant 1 − which showed the lowest IMC1a protein level − in direct comparison with the control parasite line IMC1a/mCherry-WT, and normalised against the reference tubulin 1 gene (*tub1*). Primers for *imc1a* amplified a mRNA-specific 2.4 kb product, and a gDNA-specific 2.8 kb product (due to introns), while primers for *tub1* mainly amplified a mRNA-specific 0.35 kb product (the product amplified from gDNA is 1.0 kb due to introns [7]) (Fig. 3B). Measured band intensities normalised against the reference *tub1* gene showed that IMC1a/mCherry-Mutant 1 oocysts contained approximately 1.5-fold more *imc1a* mRNA than its WT counterpart. Accordingly, the reduced levels of IMC1a present in Mutant 1, and by analogy in Mutant 2, are unlikely to be caused by a reduced *imc1a* gene expression at the transcription level.

### 3.2 The terminal cysteine motifs of IMC1a affect sporozoite shape

**Fig. 4:**
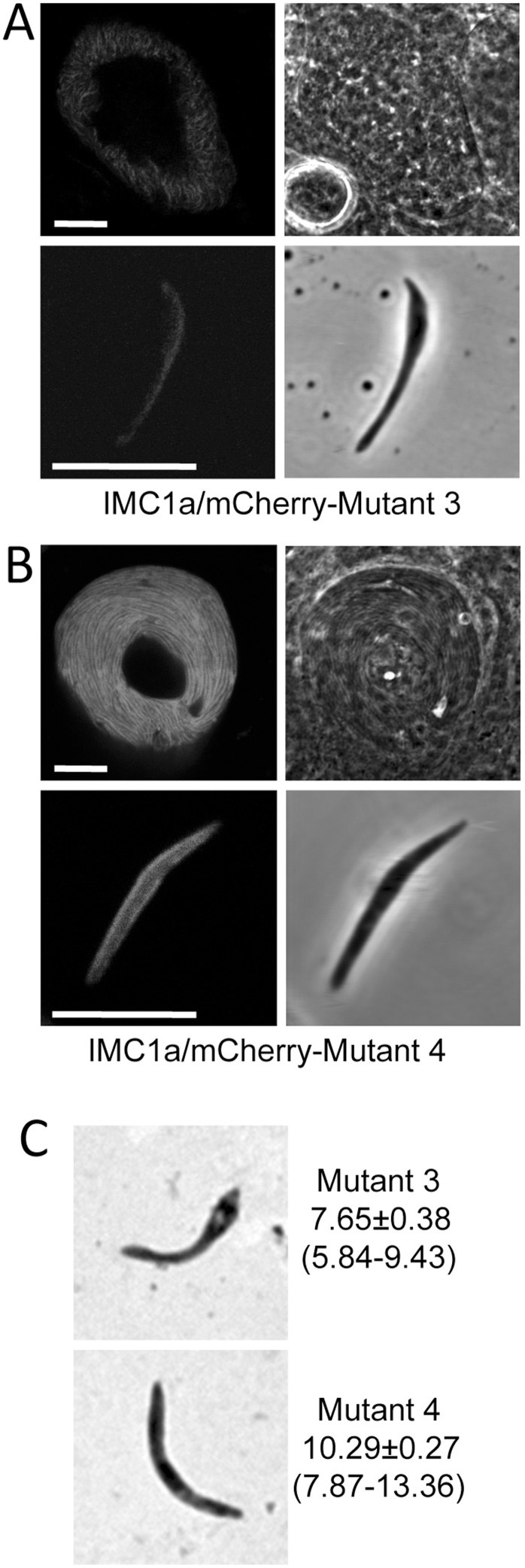
The double cysteine contributes chiefly to the function of the cysteine motif. Confocal fluorescence and brightfield images of a sporulating oocyst and sporozoite of IMC1a/mCherry-Mutant 3 (**A**) and Mutant 4 (**B**). Scale bars represent 10 μm. **C**: Representative Giemsa-stained sporozoite images of Mutants 3 and 4. Numbers give mean ± sem footprint measurements in μm^2^ and numbers in brackets give footprint range in μm^2^ (n = 27).

Microscopic examination of sporozoites from IMC1a/mCherry-Mutant 1 and Mutant 2 indicated that they had an abnormal shape (Fig. 2). Although this shape was reminiscent of the shape of IMC1a null mutant sporozoites, it appeared less severe than is the case after a complete knockout of IMC1a expression (Fig. 2C). Because of the variable and irregular shapes of the mutant sporozoites, their size was difficult to define by linear measurements of length and width. Thus, we developed a more sensitive measure of the observed changes in sporozoite morphology by determining their ‘footprint', which gives a quantitative measure of the sporozoite's size independent of its shape. Footprint data were collected by measuring the surface area occupied by Giemsa-stained sporozoites dried onto microscope slides, and showed that KO sporozoites were on average significantly smaller than WT sporozoites (P < 0.0001, n = 27), in fact less than half the normal size (Fig. 3C). Sporozoites of Mutant 1 and Mutant 2 had intermediate sizes, both being significantly smaller that WT sporozoites (P < 0.0001, n = 27), but significantly larger than KO sporozoites (P < 0.0001, n = 27) (Fig. 4C). Mutant 1 sporozoites were on average smaller than those of Mutant 2, albeit the differences were not statistically significant (P = 0.07, n = 27) (Fig. 3C). These observations demonstrate that mutations of the terminal cysteine motifs of IMC1a cause intermediate phenotypes with regards to sporozoite shape and size, compared to null mutants. We did not observe discernible shape/size differences between midgut- and salivary gland-derived sporozoite populations within our mutant lines (data not shown, and Fig. S2), pointing to similar infectivity levels throughout the population irrespective of sporozoite size/shape.

### 3.3 The terminal cysteine motifs of IMC1a affect sporozoite infectivity

We analyzed the effects of the cysteine mutations on sporozoite infectivity by infecting *Anopheles stephensi* vector mosquitoes. The reference parasite line IMC1a/mCherry-WT gave rise to high numbers of salivary gland sporozoites (Table 1) that were readily transmissible by mosquito bite. In sharp contrast, our IMC1a null mutant sporozoites were unable to reach the salivary glands in detectable numbers (Table 1) in full agreement with a previous study [5]. Salivary gland sporozoite numbers for Mutant 2 were consistently lower (3- to 4-fold) than those observed for IMC1a/mCherry-WT parasites (Table 1), pointing to a reduced infectivity to the salivary glands. Nonetheless, Mutant 2 sporozoites could be transmitted to mice by mosquito bite, and after transmission the phenotype of Mutant 2 remained unchanged with respect to fluorescence level, sporozoite size and infectivity (data not shown). In contrast to Mutant 2, salivary gland sporozoite numbers for Mutant 1 were markedly reduced ( > 10-fold) (Table 1), and we were repeatedly (n = 5) unable to transmit this parasite by mosquito bite. The lack of mosquito transmission of Mutant 1 could be caused by the low numbers of salivary gland sporozoites, or by a reduced sporozoite infectivity to the mouse, or a combination of both. Because of the low numbers of salivary gland sporozoites that could be obtained from Mutant 1-infected mosquitoes, sporozoite infectivity to the mouse by needle injection was not assessed. Nonetheless, the combined results demonstrate that IMC1a/mCherry-Mutant 1 and Mutant 2 possess intermediate phenotypes with regards to sporozoite infectivity. These infectivity data are consistent with and supported by the other phenotypes reported in this paper (IMC1a expression level, sporozoite shape and size).

### 3.4 Properties of the carboxy-terminal cysteine motif are determined by the di-cysteine

In contrast to the N-terminal cysteine motif of IMC1a, where all three cysteine residues are predicted to be palmitoylated (Fig. 1), within the C-terminal cysteine motif the di-cysteine, but not the single cysteine, are predicted to be palmitoylated (Fig. 1). If this prediction is accurate and palmitoylation were indeed key to the role of the terminal cysteine motifs, then substitution of the single cysteine of the C-terminal motif should have little effect on IMC1a stability and function, while substitution of the di-cysteine should resemble the Mutant 2 phenotype. To test this hypothesis, we generated two more mutants of the carboxy-terminal cysteine motif in IMC1a, in which either its di-cysteine (named IMC1a/mCherry-Mutant 3) or its single cysteine (named IMC1a/mCherry-Mutant 4) were substituted (Fig. S1). Subsequent phenotypic assessment revealed that Mutant 4 parasites were indistinguishable from their WT counterparts displaying bright fluorescence that was concentrated at the cortex of normal shaped sporozoites (Fig. 4B). By contrast, Mutant 3 oocysts and sporozoites displayed markedly lower fluorescence levels (Fig. 4A) and were indistinguishable from those of Mutant 2. Footprint measurements confirmed that the average size of Mutant 4 sporozoites was similar to that of IMC1a/mCherry-WT sporozoites (P = 0.99, n = 27) (Fig. 4C), while Mutant 3 sporozoites were indistinguishable from those of IMC1a/mCherry-Mutant 2 in terms of shape and size (P = 0.38, n = 27) (Fig. 4C). In terms of infectivity, too, Mutant 4 resembled its WT counterpart, while Mutant 3 behaved like Mutant 2 (Table 1). These data show that the double cysteine (CC) contributes primarily to the properties of the carboxy-terminal cysteine motif, as evidenced by the same wildtype phenotypes of the IMC1a/mCherry-WT (CC⋯C) and Mutant 4 (CC⋯A) parasite lines, as well as the same intermediate phenotypes of the IMC1a/mCherry-Mutant 3 (LE⋯C) and Mutant 2 (LE⋯W) parasite lines. These data are consistent with the hypothesis that the di-cysteine is most likely a palmitoylation site, as predicted (Fig. 1). Moreover, the single cysteine within the same motif is not required for this lipid modification to occur.

## 4 Discussion

In this study we have employed red fluorescent protein tagging in transgenic *P. berghei* parasites to study the function of the terminal cysteine residues of the alveolin IMC1a. The results obtained confirm the subcellular localisation and expression of IMC1a in the SPN of sporozoites, and show that mutagenesis of its conserved terminal cysteine motifs results in decreased protein stability causing markedly reduced levels of IMC1a protein, but not *imc1a* transcript, in the parasite. This, in turn, affects sporozoite shape, size and infectivity. We did not observe sporozoites with high (WT) levels of fluorescence in our mutant sporozoite populations. Assuming that IMC1a expression was not affected by the cysteine mutations, this suggests that the reductions in IMC1a levels occurred soon after translation and possibly before IMC1a recruitment to the pellicle.

One possible explanation for the alveolin instability is that the removal of cysteines in the mutant proteins has changed their ability to form specific sulphur bridges, leading to a degree of misfolding and degradation. Arguing strongly against this concept, however, is the localisation of IMC1a in the cytoplasm, a reducing environment that disfavours formation of intra- or intermolecular sulphur bridges [20]. A more likely explanation for the observed alveolin instability is that the removal of cysteine residues in IMC1a adversely affected the ability of this protein to be palmitoylated. S-palmitoylation is a post-translational thioester linkage of the 16-carbon fatty acid palmitate to cysteine residues that plays key roles in protein traffic, localisation, interaction and stability [21]. The conserved terminal cysteine motifs of the *Plasmodium* alveolins are predicted with high confidence to constitute palmitoylation sites (Fig. 1). Combined data from various *Plasmodium* proteomics studies reveal that at least six alveolins are detected in blood stage parasites [22], [23], [24], [25], [26], [27], [28], [29]. Among these six only two, IMC1c and IMC1g, possess conserved terminal cysteine motifs (at the carboxy- and amino-terminus, respectively) (Fig. 1). Only IMC1c and IMC1g were detected in the *P. falciparum* blood stage palmitome [30], providing compelling experimental support for a link between these motifs and palmitoylation. Palmitoylation is known to affect protein stability [21], and indeed other known palmitoylated proteins associated with the parasite pellicle (e.g. *Pf*GAP45 and *Pf*MTIP) have been reported to become unstable and degraded when palmitoylation was interfered with using palmitoylation inhibitors or mutagenesis of palmitoylation sites [30]. Unfortunately, the relatively small numbers of sporozoites that can be obtained from mosquitoes − a major impediment to biochemical analysis [31] − combined with the presence of a second palmitoylation site at the other end of the protein, and the highly reduced levels of IMC1a in Mutants 1 and 2, made it impossible to demonstrate that the terminal cysteine motifs of IMC1a are palmitoylation sites as predicted. Nonetheless, a molecular role for the terminal cysteine residues of IMC1a as substrates for palmitoylaton seems likely, and indeed is supported by our observation that mutagenesis of the C-terminal single cysteine residue, not predicted to be palmitoylated (Fig. 1), has no discernible effect on IMC1a stability and function (Fig. 4). Palmitoylation of alveolins is thought to promote their association with the IMC via lipid anchoring into the inner membrane [14]. Reductions in IMC1a palmitoylation caused by the cysteine mutations could adversely affect this process and/or protein folding or interaction, which in turn could result in instability and degradation [32].

Gene disruption studies of different *P. berghei* alveolins have revealed very similar loss-of-function phenotypes adversely affecting morphology, motility and tensile strength [5], [8], [9], [10], [11], indicating that alveolins contribute to the function of the SPN through a similar mechanism. While co-expressed alveolins make distinct contributions to SPN function in a given zoite, these differences appear to be mainly quantitative. For example, null mutants sporozoites of the alveolin IMC1h have an abnormal shape and reduced infectivity not dissimilar from IMC1a null mutants [10]. However, in contrast to the latter, IMC1h-KO sporozoites retain infectivity to the insect’s salivary glands [11]. Re-examination of the IMC1h-KO sporozoite size with the footprint method developed here shows that they are indeed significantly smaller (P < 0.001) than their wildtype counterparts (parasite line IMC1 h/GFP), but also significantly larger (P < 0.0001) than IMC1a null mutant sporozoites (footprint IMC1 h/GFP: 10.51 ± 0.41 μm^2^; IMC1h-KO: 7.98 ± 0.44 μm^2^). These observations reveal a correlation between sporozoite size and infectivity, which is corroborated by the results of the current IMC1a study.

The intermediate phenotypes of IMC1a/mCherry-Mutant 1 and Mutant 2 with regards to sporozoite shape, size and infectivity (Fig. 2, Fig. 3, Table 1) indicate that the heavily depleted amount of IMC1a protein in their sporozoites remains at least partly functional, possibly because of a degree of functional redundancy between the amino- and carboxy-terminal cysteine motifs. The low levels of IMC1a:mCherry in these mutants did not allow a definitive allocation of the fusion protein in the sporozoite SPN by fluorescence microscopy (Fig. 2). However, complete failure to localise to the functional site would be expected to result in a null mutant phenotype, which is clearly not the case. In further support for this notion, subcellular localisation of *Pf*GAP45 lacking its palmitoylation site was shown to be unaffected, remaining at the IMC of developing merozoites [30]. Our Mutant 1 and Mutant 2 parasite lines are in effect IMC1a knockdowns, and combined with our observations that the severity of the phenotypes correlates well with the levels of IMC1a found in the parasite, this strongly indicates that IMC1a facilitates sporozoite morphogenesis and infectivity in a dose-dependent fashion. This demonstrates for the first time a link between the amount of alveolin in the zoite and its infectivity. Accordingly, SPN function in the cell would be governed not only by the repertoire of co-expressed alveolins, but also by the level of their expression. Fitting with the nature of structural proteins, this finding suggests that the relative abundance of an alveolin in the cell may determine its relative contribution to the cortical cytoskeleton. In support of this concept, it is perhaps not a coincidence that knockout of the ookinete-expressed alveolin IMC1d has no detectable phenotype, and also has the lowest expression level of all ookinete-expressed alveolins examined by us [7], [9], [10], [16]. Just a fraction of IMC1a (10–21% of WT level in Mutant 1 and Mutant 2) is sufficient to partially restore the phenotype of IMC1a null mutant parasites (Fig. 3C, Table 1), which points to a non-linear correlation between alveolin level and phenotype, possibly due to the complexity of interactions with other alveolins and SPN components. The loss of infectivity of IMC1a null mutants is attributable to not just the reduced size and abnormal shape of the sporozoites, but also to accompanying reductions in motility (approximately 5-fold) and tensile strength of the sporozoites [5]. By inference, the markedly reduced levels of IMC1a present in sporozoites of Mutant 1 and Mutant 2 (Fig. 3A) would result in reduced levels of motility and tensile strength, contributing to the observed losses in sporozoite infectivity in these parasite lines. Mutation of the N-terminal cysteine motif of IMC1a (Mutant 1) causes more severe phenotypes than substitution of the C-terminal cysteine motif (Mutant 2) with regards to IMC1a stability (Fig. 3A) and infectivity to the salivary glands (Table 1). This difference could reflect quantitative differences in palmitoylation at the N- and C-terminus, as predicted (Fig. 1). Alternatively, the cysteine motifs may possess additional roles besides acting as palmitoylation sites, which could be different between the N- and C-terminus.

*Tg*IMC1, the *T. gondii* orthologue of *Plasmodium* IMC1a, contains similar N- and C-terminal terminal cysteine motifs [4], [5]. However, the C-terminal cysteine motif of *Tg*IMC1 has several additional downstream cysteine residues and is not predicted to be palmitoylated with the method used here. Studies on *Tg*IMC1 revealed a specific proteolytic cleavage near the carboxy-terminus of the protein upstream of its cysteine motif [33]. This cleavage coincides with the maturation of young parasites with a detergent-labile SPN into mature parasites with a detergent-resistant SPN. Ablation of this cleavage through mutagenesis of certain cysteines prevented the SPN to mature and become detergent-resistant [33]. In the case of *P. berghei* IMC1a, there is little evidence for a similar cleavage event. Western analysis of our parasite lines expressing IMC1a fused to a carboxy-terminal mCherry tag shows that sporozoites contain predominantly full-length IMC1a:mcherry fusion product (Fig. 2B), and that if such a cleavage occurred in *Plasmodium* it would at best affect a very small proportion of the protein. It cannot be ruled out that the mCherry tag stopped the protease responsible from reaching the C-terminal cleavage site, preventing the IMC1a cleavage in our parasite lines. Arguing against this possibility, however, is the fact that mCherry tagging of IMC1a did not affect its subcellular localisation or cause a detectable phenotype, which indicates that IMC1a function was not affected. The apparent absence of this cleavage in IMC1a is more likely to reflect specific differences between *Toxoplasma* and *Plasmodium*. The formation of *Plasmodium* sporozoites within a protective oocyst capsule may have eliminated the need to develop within the protection of mechanically stable and rigid mother cell as is the case in *Toxoplasma*.

## Figures and Tables

**Table 1:** Effects of IMC1a mutations on *Plasmodium berghei* parasite development in *Anopheles stephensi* mosquitoes.

Exp.	Parasite line IMC1a/mCherry-	Mean ± sem oocyst number per infected mosquito (n)^a^	Mean salivary gland sporozoite number per infected mosquito^b^
1	WT	39 ± 7 (20)	10,900
	KO	47 ± 8 (20)	0

2	WT	104 ± 42 (10)	6600
	Mutant 1	97 ± 32 (10)	110
	Mutant 2	98 ± 27 (10)	2800

3	Mutant 1	94 ± 13 (20)	260
	Mutant 2	133 ± 24 (20)	3100

4	WT	109 ± 31 (10)	4900
	Mutant 1	141 ± 50 (10)	220
	Mutant 4	120 ± 44 (10)	5600

5	Mutant 2	34 ± 9 (20)	2000
	Mutant 3	36 ± 9 (20)	2800

6	WT	160 ± 33 (10)	16,600
	Mutant 2	192 ± 30 (10)	3900

7	Mutant 1	47 ± 11 (10)	330
	Mutant 4	78 ± 25 (10)	9900

a n = Number of insects analysed.

b The same insects were analysed for both oocyst and sporozoite numbers. Oocyst and sporozoite numbers are therefore directly linked, eliminating much of the variability associated with sample size. Oocyst counts were done on individual midguts, sporozoite counts were done on pooled salivary glands from n insects.
