# Supplementary material for: The Plasmodium alveolin IMC1a is stabilised by its terminal cysteine motifs and facilitates sporozoite morphogenesis and infectivity in a dose-dependent manner
Source: Mol Biochem Parasitol. 2017 Jan;211:48–56. doi: 10.1016/j.molbiopara.2016.09.004 (PMC5223771; doi:10.1016/j.molbiopara.2016.09.004)
Supplement: Supplementary file 1 [file mmc1.pdf]

Supporting information

The *Plasmodium* alveolin IMC1a is stabilised by its terminal cysteine motifs and facilitates sporozoite morphogenesis and infectivity in a dose-dependent manner.

Fatimah S. Al-Khattaf, Annie Z. Tremp, Amira El-Houderi, and Johannes T. Dessens

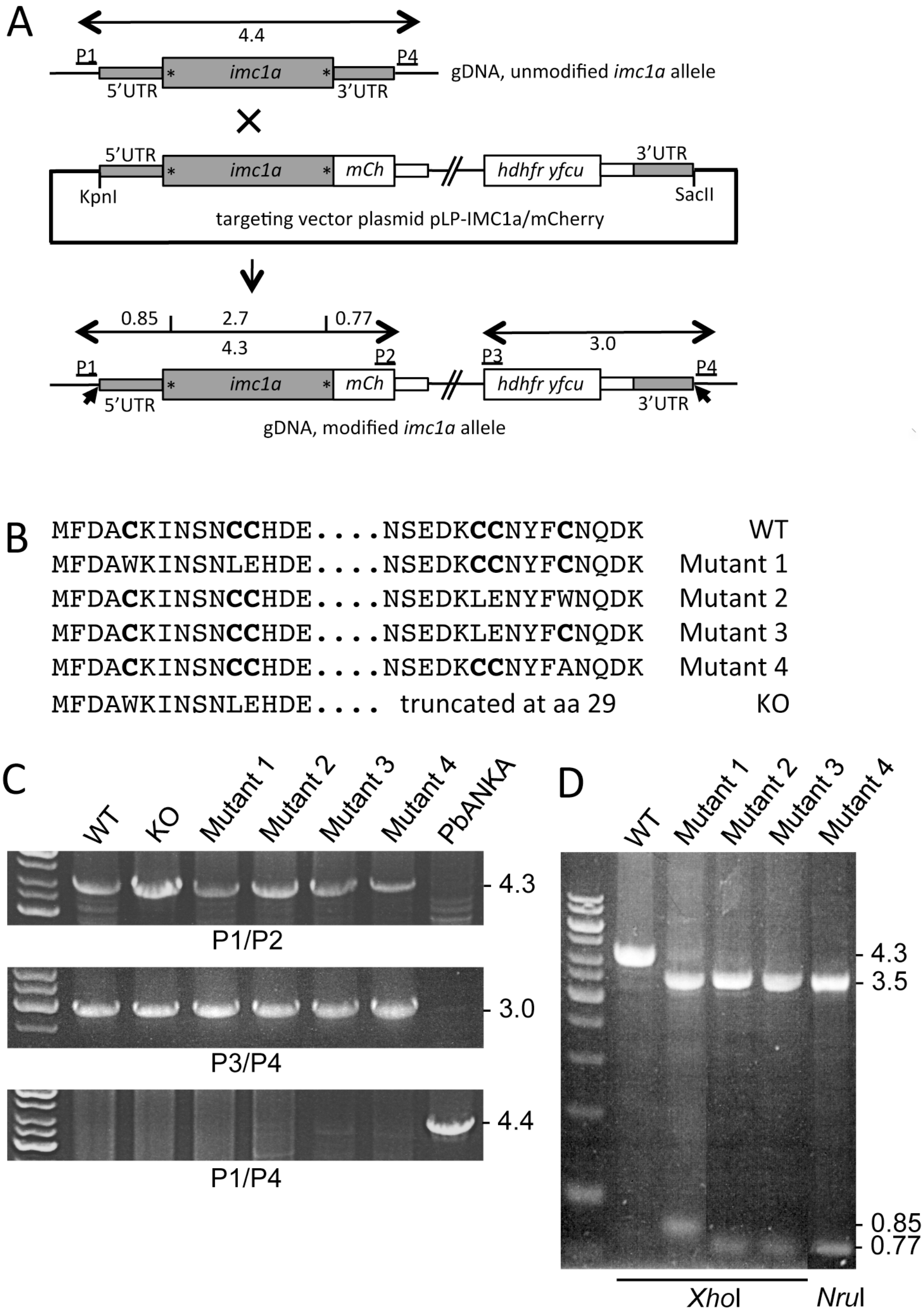

Fig. S1 Generation and genetic analyses of mCherry-tagged IMC1a parasite lines. **A**: Targeting strategy for the mCherry tagging of IMC1a via double crossover homologous recombination, showing unmodified (*top*) and modified (*bottom*) *imc1a* alleles, a well as the targeting vector pLP-IMC1a/mCherry (*middle*). The *pbimc1a* gene is indicated with coding sequence (wide grey bars, introns not shown) and 5' and 3' untranslated regions (UTRs) (narrow grey bars). Also indicated are the relative positions of the terminal cysteine motifs (asterisks), the mCherry module (*mCh*); the hDHFRyFCU selectable marker gene cassette (*hdhfr yfcu*); the generic 3'UTR from *pbdhfr* (narrow white bars); and primers used for diagnostic PCR amplification (P1-P4). Primer P1 and P4 sequences are not present within the targeting vector. Arrowheads mark 5' and 3' integration sites. Also shown are the relative positions of *Kpn*I and *Sac*II restriction sites used to remove the plasmid backbone from the targeting vector. **B**: Amino acid sequences of the amino- and carboxy-terminal ends of IMC1a in parasite lines IMC1a/mCherry-WT (wildtype IMC1a sequence, but with mCherry tag), IMC1a/mCherry-Mutant 1, IMC1a/mCherry-Mutant 2 and IMC1a/mCherry-KO (null mutant). The conserved cysteines are marked in bold. **C**: PCR with primer pair P1/P2 across the 5' integration site diagnostic for the presence of the mCherry-tagged *pbimc1a* alleles (*top*) amplifies an expected 4.3kb fragment; PCR with primer pair P3/P4 across the 3' integration site diagnostic for the presence of the *hdhfr yfcu* selectable marker allele (*middle*) amplifies an expected 3kb fragment; and PCR with primer pair P1/P4 diagnostic for the presence/absence of the unmodified *imc1a* allele (*bottom*) amplifies an expected 4.4kb fragment in parental (PbANKA) parasites. **D**: Restriction enzyme digestion (*Xho*I or *Nru*I) of 4.3kb amplicons from **C,** diagnostic for the presence of the desired cysteine motif mutations. Cleavage products of 3.5kb and either 0.85kb (Mutant 1), or 0.77kb (Mutants 2-4) are visible except for the non-mutated version of *imc1a::mcherry* in parasite line IMC1a/mCherry-WT.

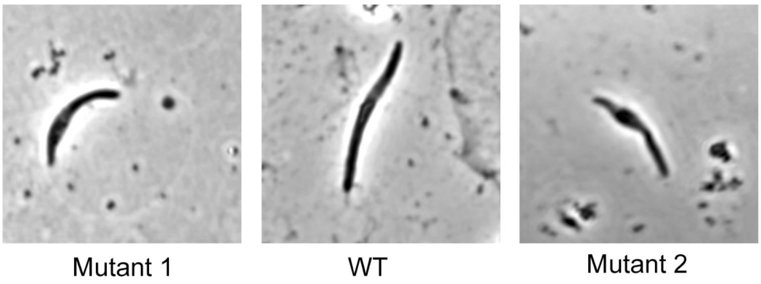

Fig. S2 Representative Giemsa-stained midgut sporozoite images from parasite lines IMC1a/mCherry-WT and Mutants 1-2.
